# Editorial: Advancements in injury rehabilitation and return-to-sport practices

**DOI:** 10.3389/fspor.2026.1795108

**Published:** 2026-02-24

**Authors:** Anna Christakou, Konstantinos Fousekis, Maria Constantinou

**Affiliations:** 1 Laboratory of Biomechanics, Department of Physiotherapy, University of Peloponnese, Sparta, Greece; 2 Department of Physiotherapy, University of Patra Greece, Patra, Greece; 3 Physical Activity, Sports and Exercise Research Theme, Faculty of Health, Southern Cross University, Gold Coast, QLD, Australia

**Keywords:** editorial, prevention, readiness, return-to-play, sport

Sports injury rehabilitation and return to sport (RTS) is multi-faceted and how it is addressed can profoundly influence an athlete's long-term performance and career longevity. Optimal sports injury rehabilitation involves not only incorporating the biological and physical aspects but also psychological, social, and contextual factors. These factors collectively influence an athlete's recovery trajectory, injury prevention and timely return to sport. Findings from studies included in this research topic on *Advancements in Injury Rehabilitation and Return-to-Sport Practices* highlighted rehabilitation strategies that encompasses the whole athlete, to support a safe and sustainable return to sport.

Both research evidence and clinical experience consistently demonstrate that premature return to sport following musculoskeletal injury is among the strongest predictors of re-injury. While a rapid comeback may satisfy competitive demands and external pressures, it may compromise tissue healing, neuromuscular control, and psychological readiness—factors essential for peak performance and injury prevention. Athletes require structured and progressive injury rehabilitation and re-integration into training and competition, accompanied by ongoing monitoring of workload, recovery status, psychological readiness and symptom response for optimal return to sport and prevention of re-injury.

## Sports injury rehabilitation should be sports- and condition-specific, address muscle and functional performance, and consider the joint health, while tissue type and mechanism should be considered in return to sport and injury prevention strategies

In the following section we outline an overview of recent research on common conditions such as patellofemoral pain (PFP), anterior cruciate ligament reconstruction (ACLR) and the athletic hip, which provides valuable insights into condition-specific rehabilitation approaches and recovery trajectories, and we outline an overview describing the importance of incorporating injury tissue type and mechanism into prevention strategies.

Patellofemoral pain remains one of the most common injuries among runners, with muscle strengthening being an important component for stage-specific rehabilitation and return to sport. Evidence from recent research in PFP by He et al. indicates that quadriceps strength varies according to the duration of symptoms, with individuals exhibiting reduced strength in the short-term (<3 months) but not in the long-term (>12 months). Interestingly, He et al. found that individuals with long-term symptom duration demonstrated comparable strength levels to healthy controls. In contrast, hamstring strength, hamstring-to-quadriceps ratios, and muscle symmetry appear largely unaffected by PFP duration (He et al.). Although limitations exist—particularly the use of single-velocity isokinetic testing and concentric-only contractions—these findings enhance our understanding of strength adaptations across PFP stages and may guide more targeted, stage-specific rehabilitation strategies.

Although adjunct rehabilitation interventions such as blood flow restriction training (BFRT) may have previously demonstrated mixed results in muscle strength improvements following anterior cruciate ligament reconstruction (ACLR), according to findings by Barzyk et al., when BFRT is combined with low-load muscle strengthening protocols no differences were seen for knee muscle strength, range of motion, pain and function recovery in the early post-operative phase. High-quality, multicenter randomized controlled trials are thus recommended to further clarify the mid- and long-term role of BFRT in postoperative rehabilitation of ACLR.

Hip and groin injuries continue to present rehabilitation and RTS challenges. A recent opinion review on the athletic hip and key elements required for RTS by Bizzini et al. highlights the importance of incorporating the hip joint health status and muscle strength testing into the rehabilitation and RTS continuum. Discussing the hip joint health with the athlete and providing guidance into the expectations is paramount. The authors also recommend as part of the rehabilitation phase greater emphasis is placed on valid and reliable routine hip strength testing and sport-specific strengthening that incorporates both isolated hip exercises in all planes and multi-segment hip exercises (Bizzini et al.). Functional performance assessments are also recommended during both the rehabilitation phase and ongoing training for return to sport. Further, stabilization exercises should be integrated into warm-up routines or dedicated training sessions. With a high rate of hip and groin re-injuries, the authors stress there is a need for effective secondary and tertiary prevention strategies (Bizzini et al.).

Addressing the injury by tissue type and mechanism during the rehabilitation process may enable implementation of better injury preventative strategies to reduce re-injury risks. A recent narrative review on a synthesis of football injury types and prevention strategies by Zeng et al. proposed a theoretical basis for understanding injuries in athletes. As proposed by Zeng et al., skeletal muscle injuries, including muscle fiber and tendon injuries, may be mitigated through eccentric strength training, whereas joint injuries such as ligament damage and muscle imbalances require emphasis on neuromuscular control. Degenerative injuries demand systematic, often long-term management, with surgical intervention considered when appropriate and followed by individualized rehabilitation. Accidental injuries, including concussions and fractures, can be reduced through protective equipment, rule modifications, structured training programs and enhanced safety education. The authors conclude that by ensuring injury prevention strategies are mechanism-specific, injury risk in sports may be reduced (Zeng et al.).

## Psychological readiness is central to successful return to sport

Equally important—but often underemphasized—is the psychological dimension of RTS. In the following section we outline findings from two recent studies that emphasized the significance of psychological considerations in return to sport and injury prevention.

A recent narrative review on the psychological dimensions of sports injury risk by Johnson and Ivarsson presented contemporary models describing overuse and traumatic injuries as different multifactorial phenomena shaped by various intrapersonal, interpersonal, and sociocultural factors. The authors describe impaired neurocognitive functioning, academic stress, and dysfunctional coach–athlete relationships to be associated with elevated injury risk (Johnson and Ivarsson). Practical strategies proposed by the authors to reduce injury risk include fostering supportive athlete–staff relationships, offering confidential psychological support services, and embedding health education and autonomy-building practices into daily training environments (Johnson and Ivarsson). Consequently, integrating biopsychosocial frameworks with multidisciplinary collaboration between sports psychologists, medical professionals and coaches is recommended by the authors to support effective injury prevention and return to sport practices.

Psychological readiness has also emerged as a critical determinant of successful RTS. A study by Butler et al. recently reported higher psychological readiness associated with reduced kinesiophobia among adolescents and young adults 6–12 months following ACLR, although nearly half of athletes from both groups reported poor psychological readiness. These findings underscore the need for targeted psychological interventions during rehabilitation to improve psychological readiness for return to sport. The authors recommend future research should further explore the influence of sex and age on emotional responses, confidence, and risk appraisal, while accounting for variability in rehabilitation protocols, surgical details, and actual RTS outcomes (Butler et al.).

In conclusion, while sports injuries may be inevitable, recurrent injuries are not. We presented evidence from six recent articles included in this topic on knowledge in injury rehabilitation and return to sport practices, advocating for a holistic rehabilitation model that integrates physical, psychological, and social factors alongside evidence-based physiotherapy. Such an approach not only supports a successful return to sport, but also reduces the risk of long-term re-injury, psychological distress, and performance decline. The ultimate goal of rehabilitation is not merely returning to sport—but returning well, and with injury-free participation and performance enhancement over time.

